# Supplementary material for: Are carbon emissions trading and green financial instruments synergistic? -Comprehensive quantitative research based on content analysis
Source: PLoS One. 2024 Mar 7;19(3):e0298601. doi: 10.1371/journal.pone.0298601 (PMC10919601; doi:10.1371/journal.pone.0298601)
Supplement: S1 Data — (ZIP) [file pone.0298601.s001.zip › Data/地级市Z_F工作报告.zip!/╣π╬≈/│τ╫≤╩╨/└┤╫╘╣1⁄2╓┌║┼í╛┬φ┐╦╩2╛▌═°í┐_2019.docx]

各位代表：

　　现在，我代表市人民政府，向大会报告政府工作，请予审议，并请市政协委员和其他列席会议的同志提出意见。

一、 2018年工作回顾

　　2018年是贯彻党的十九大精神的开局之年，是我国改革开放40周年，是自治区成立60周年，是崇左建市15周年，也是做好“两篇大文章”、打好“四大攻坚战”三年行动计划的收官之年。一年来，面对错综复杂的形势，在自治区党委、政府和市委的正确领导下，我们坚持以习近平新时代中国特色社会主义思想为指导，深入学习贯彻党的十九大和十九届二中、三中全会精神，坚持稳中求进工作总基调，按照高质量发展的要求，保持战略定力，坚定不移做好“两篇大文章”、打好“四大攻坚战”，统筹推进稳就业、稳金融、稳外贸、稳外资、稳投资、稳预期工作，促进经济社会发展稳中有进、稳中向好、稳中提质，圆满完成了市四届人大三次会议确定的目标任务，朝着与全国全区同步全面建成小康社会、建设祖国南疆繁荣稳定和谐安宁示范区的目标迈出了坚实步伐。

　　一年来，我们主要做好以下十个方面的工作：

　　（一）稳增长、强落实，经济向高质量发展迈进。坚持把稳增长作为全年工作的重中之重，全面贯彻国家、自治区各项政策措施，狠抓工作落实，强化招商落地开工、项目竣工投产、企业上规入统“三个终端见成效”，实现了经济社会持续健康发展。初步预计，全市地区生产总值1002亿元，首次突破1000亿元大关，同比增长10.8%，增速首次排全区首位；规模以上工业总产值1088亿元，首次突破1000亿元大关，增长18%，增速排全区第3位；规模以上工业增加值369亿元，增长16%，增速排全区第2位；固定资产投资增长18%，排全区第2位；外贸进出口总额1560亿元，增长12.5%，总量连续10年稳居全区第一；财政收入57.58亿元，增长4.2%，其中税收收入增长11.1%；社会消费品零售总额163亿元，增长11.7%，增速排全区第3位。就业总体稳定，全市城镇登记失业率2.35%，比年度目标控制数低1.85个百分点。城乡居民收入稳步增长，城镇居民人均可支配收入30830元，增长7%，农村居民人均可支配收入11946元，增长10%，增速均排全区前列。县域经济加快发展，扶绥县、江州区荣获2017年广西科学发展进步县称号。总体看，全市经济运行好于预期，向高质量发展稳步迈进。

　　（二）抓开放、促合作，口岸经济提量增效。坚持开放发展主基调，加快建设开放合作平台，凭祥重点开发开放试验区建设若干政策落地实施，凭祥综合保税区被评为全国第二批国家级示范物流园区、全国优秀物流园区。凭祥市推进边贸转型升级经验做法获国务院通报表扬。边境贸易扩量提质，我市位列2017年中国外贸百强城市第28位。口岸加工业壮大发展，新增规模以上口岸贸易加工企业22家，累计达65家，口岸贸易加工总产值预计达100亿元，增长41.9%，“通道经济”加快向“口岸经济”发展。沿边金融综合改革创新推进，率先开展中越人民币现钞跨境调运，跨境人民币累计结算量连续8年居全区第一。跨境物流体系进一步完善，崇左（凭祥）列为陆上边境口岸型国家物流枢纽承载城市，中泰产业园公用型保税仓获批设立，新开通中欧班列（凭祥—河内）跨境集装箱直通运输班列。先行先试取得实质性进展，友谊关口岸在全国边境口岸率先实现周末、节假日正常通关，是全国首个实现全信息化智能通关的沿边口岸；中越跨境劳务合作试点工作加快实施，累计登记跨境务工人员13.7万人次。互联互通建设不断加强，爱店口岸升格为一类口岸正式对外开放，水口口岸扩大开放为国际性口岸通过自治区验收，凭祥铁路口岸升级改造工程（一期）竣工启用，南宁至崇左城际铁路等21个项目纳入广西“一带一路”百项重点工程。国际“朋友圈”不断扩大，友好城市增加到28个，荣获2016—2017年度“国际友好城市交流合作奖”。

　　（三）抓特色、创品牌，文化旅游提速发展。全域旅游加快推进，7个县（市、区）均入选国家级或自治区级全域旅游示范区创建单位，龙州县成功创建广西特色旅游名县，宁明县成功创建自治区级全域旅游示范区。特色品牌进一步打响，德天跨国瀑布成功创建国家5A级旅游景区，我市成为广西第三个拥有国家5A级景区的设区市，新增6家4A级旅游景区、2家四星级旅游饭店、5家四星级广西乡村旅游区（农家乐）。旅游重点项目建设取得新进展，引入中旅集团运营德天跨国瀑布和左江花山岩画景区，左江花山音画夜游、实景演出实现试运营，一批旅游民宿项目建设稳步推进。跨境旅游加快推进，中越广西—谅山跨境自驾车旅游常态化开通，中越德天—板约瀑布跨境旅游合作区加快建设。旅游配套设施建设不断加强，南友高速公路花山互通建成通车，新建改建旅游厕所43座。扶持政策有效落实，一批旅游景区、星级旅游饭店等获得奖励资金共1426万元。旅游商品实现新发展，近百种旅游商品正式上线销售。旅游宣传实现新突破，参加中央电视台《魅力中国城》第二季竞演以年度总成绩第一名荣膺“十佳魅力城市”榜首，充分绽放了山水崇左、甜蜜边关、魅力壮乡的独特风采，极大增强了崇左人的文化自信和家乡认同感、荣誉感、归属感。文旅节会多点开花，成功举办广西第十四届少数民族传统体育运动会、“壮族三月三·崇左花山国际文化旅游节”、中国文联文艺志愿服务团“送欢乐下基层”走进崇左慰问演出和国际体育旅游嘉年华等活动。崇左旅游知名度美誉度显著提升。预计全年接待游客3600万人次，增长39.5%；实现旅游总消费340亿元，增长38.9%。

　　（四）抓转型、促提升，产业结构优化升级。围绕工业发展主战场，加快工业转型发展，新增木材加工、口岸加工、化学工业3个百亿元产业，全市百亿元产业达5个，产业基础明显夯实。企业效益稳步提升，全市规模以上工业企业主营业务收入增长14.1%，工业企业增值税增长12.9%。工业企业规模不断扩大，新增投产入规工业企业46家，新增数量排全区第2位。园区建设稳步推进，中泰产业园、海峡两岸产业合作区崇左产业园、广西·中国糖业产业园等园区基础设施不断完善，新建成标准厂房98.7万平方米，企业累计入驻率70.5%，全市工业园区总产值 775亿元，增长11%。制糖企业整合重组有新进展，大新永鑫糖业集团与湘桂糖业集团实现深度合作，广西糖业集团与大新世纪飞龙糖业公司重组基本完成。重点产业项目建设实现新突破，东亚糖业循环经济综合利用、湘桂乐斯福酵母扩建一期、中信大锰锰酸锂技改一期等项目竣工投产，稀土新材料产业园、龙州生态氧化铝等项目加快建设，南国铜业项目一期实现点火，新动能加快成长。现代农业稳中求新，粮食生产功能区和糖料蔗生产保护区划定工作基本完成，预计粮食总产量52.61万吨，新建“双高”基地65.18万亩，新增现代特色农业示范区（园、点）411个、牛羊生态养殖小区（场）82个、广西畜禽现代生态养殖场76家、农业企业153家、农民合作社538个、家庭农场 111家、“三品一标”认证农产品14个。现代服务业加快发展，成立全区首个服务业发展办公室，新增入库服务业企业（含大个体）205家。我市被商务部、国台办列为国家第三批两岸冷链物流产业合作试点城市，第三产业产值372亿元，增长10.2%，拉动GDP增长4个百分点。实施创新驱动战略取得新成效，市高新技术产业开发区通过自治区评估，科技成果转化率稳步提升。三次产业结构优化调整为 18.9：43.9：37.2。

　　（五）抓精准、提质量，脱贫攻坚再创佳绩。以专业扶贫工作队为抓手，全力打好产业扶贫、村集体经济、易地扶贫搬迁、基础设施建设、粤桂扶贫协作“五场硬仗”，多措并举推进农村全面脱贫攻坚战，脱贫质量不断提升。预计全年减贫104541人，103个贫困村脱贫出列，贫困发生率由2015年底的18.16%下降到3.35%，龙州县成功脱贫摘帽，打响广西国家扶贫开发重点县脱贫摘帽第一炮。创新精准帮扶模式，组建精准帮扶网格化专业扶贫工作队，选派村党组织第一书记841名、工作队员2138名、科技特派员152名，提升了精准帮扶工作水平。推动“扶贫车间”多样化、多元化发展，累计建成各类“扶贫车间”695家，有力带动贫困群众在家门口实现就业，人均月增收1900元以上。实施村集体经济“提速增效”工程，2018年计划脱贫出列村和109个已脱贫出列村集体经济收入全部达到3万元以上。全面完成易地扶贫搬迁任务，累计搬迁贫困人口41379人。加强与广东省江门市扶贫协作，累计获得扶贫协作资金2.1亿元，实施扶贫项目83个。“百企扶百村·万人帮万户”活动深入开展。强化扶贫惠民政策落实，控辍保学成效显著，贫困县义务教育巩固率达95%以上，创新开展健康扶贫“一站式”即时结算工作并获全区推广，完成预脱贫户危房改造4247户。加大扶贫资金精准投入，财政扶贫资金投入18.84亿元，增幅21%。扎实推进大新县、宁明县脱贫摘帽工作，目前两县均已达到国家“两不愁三保障”脱贫标准，有望实现摘帽目标。开展扶贫领域腐败和作风问题专项治理，认真抓好中央、自治区脱贫攻坚巡视反馈意见整改。

　　（六）抓统筹、促融合，城乡建设协调推进。围绕建设山水园林城市目标，统筹推进新型城镇化建设，促进城乡融合发展，全市城镇化率提高至40.5%。编制完成《崇左市城市总体规划（2017—2035年）》并获自治区政府批准实施。中心城市提升工程深入实施，崇左汽车城、华润城南综合体、环城北路等项目开工建设，七大系列65个项目已开工58个，太平古城改造、爱琴海购物公园、中心城区河湖水系连通一期工程等项目加快建设，市体育中心、崇左大桥主体工程竣工，中心城区建设完成投资91.4亿元，新增建成区面积2.62平方公里，实现大投入大变样。园林建设加快推进，金龙湖公园、东盟大道提升工程竣工使用，中心城区新增公园绿地面积120公顷。全面开展自治区文明城市创建活动，深入实施城区市容环境综合整治“百日攻坚行动”，中心城区实现“通起来”“亮起来”“绿起来”“美起来”，城市建设管理由“重建轻管”向“建管并重”转变。大县城战略有序实施，凭祥城乡一体化示范区和国家中小城市改革试点建设加快推进。全国特色小镇江州区新和镇、广西特色小镇龙州县水口镇、扶绥县山圩镇和44个乡土特色示范村屯加快建设，城镇发展牵引力进一步增强。乡村振兴战略开局良好，建立200多个项目总投资600多亿元的乡村振兴战略项目库，“旱改水”项目竣工验收30个，新增水田面积2.86万亩；美丽乡村建设扎实推进，“百村示范百村整治”工程、10个星级村、“四个好”宜居村庄建设有序实施，乡村宜居水平不断提升。打好污染防治攻坚战，生态环境持续改善，空气质量优良天数比例95.1%、综合指数排全区第3位，纳入“水十条”考核的5个断面水质和市级集中式饮用水源水质100%达标，市级集中式饮用水源地保护工作全面完成。

　　（七）抓项目、促投资，发展基础不断夯实。以“双10”重大项目建设为抓手，加快一批交通、水利等重大项目建设步伐，促进投资快速增长，增速保持全区前列。落实项目前期工作经费5.28亿元，有力保障项目前期工作开展。67项重大项目纳入自治区层面统筹推进，项目数量为历年之最。崇左港濑湍作业区一期投入运营，南宁至崇左城际铁路全线进场施工，崇左至水口高速公路、隆安至硕龙高速公路、大新德天至宁明花山一级公路等项目加快建设，左江治旱驮英水库大坝实现截流。民生基础设施不断完善，天等县正式通天然气，110千伏石林送变电工程、龙州县120兆瓦光伏项目竣工运营。融资规模稳步扩大，完成重大项目融资480.36亿元，增长12.7%；成功发行13亿元企业债券，实现资本市场直接融资零的突破。招商引资力度前所未有，实施“一把手”招商工程，大力开展产业链招商、展会平台招商、以商招商、委托招商，成功举办“创业中华—海内外华商八桂行共筑魅力崇左新发展”投资推介会等活动，全市区外招商引资项目到位资金355亿元，增长9%，其中全口径实际利用外资到位资金3.5亿美元，增长41%。

　　（八）抓改革、破难题，营商环境明显改善。围绕建设创新型政府，大力推动政府治理理念创新、服务模式创新、体制机制创新，不断释放创新创造活力和强劲动能。聚焦优化营商环境痛点难点问题，研究制定“1+N”系列配套文件。深化行政审批制度改革，全面推进“一事通办”改革，编制市县两级“一次性告知”“最多跑一次”“一次不用跑”3张清单并向社会公布，行政权力事项开通网上办理比重达88.4%，我市被列入全区一体化政务服务平台首批试点城市。深入推进“多证合一、一照一码”“证照分离”等商事制度改革，企业名称自主申报改革实现“一次不用跑”。商标品牌工作走在全区前列，注册商标数量同比增长35.2%，建立全区首个商标品牌创新创业基地，地理标志证明商标实现零的突破。服务企业水平明显提升，在全区率先成立服务企业办公室，开展“市长服务企业日”活动，执行市领导挂点服务企业机制，建立服务企业专员制度，搭建服务企业综合信息平台，覆盖市场主体32676家。出台重大项目并联审批办法，政府性投资项目审批时间由原来225个工作日压缩到56个工作日，企业投资项目审批时间由原来的205个工作日压缩到30个工作日，分别压缩75.1%、85.4%。电力体制改革成效明显，电力签约交易量38.4亿千瓦时，排全区第3位，为企业降成本2.4亿元。农村改革深入推进，农村集体产权制度改革试点工作有序开展，农村土地承包经营权证书颁发率95.4%。国企改革取得新进展，整合重组10家国有企业，组建市城市建设投资发展集团有限公司、市城市工业投资发展集团有限公司。

　　（九）办实事、惠民生，百姓福祉持续增进。全面完成十项为民办实事工程。全市民生支出212.19亿元，占一般公共预算支出的比重为82.2%。教育事业加快发展，新建、迁建、改扩建教育项目共555个，建成357个，完成投资18.59亿元。广西自然资源职业技术学院、南宁地区教育学院新校区、广西大学附属中学崇左校区等建成使用；基础教育“质量提升工程”三年行动计划圆满收官，扶绥县、江州区通过国家义务教育均衡发展评估验收，我市成为广西第三个所有县（市、区）通过国家验收的设区市；广西城市职业学院被教育部确定为全国第一批职业教育本科试点学校。健康崇左加快建设，新增2家医联体、28家医共体，在全市所有公立医疗机构药品采购中推行“两票制”，启动国家卫生城市和全国幸福家庭活动示范市创建工作，荣获“2013—2017年度全国创建‘平安医院’活动表现突出地区”称号。社会保障水平稳步提高，城乡低保生活补助标准平均每人每年提高240元，城乡居民基本医疗保险和基本养老保险参保率分别达到98.4%、97%，各项社会保险待遇发放率100%。保障性住房建设有序开展，棚户区改造基本建成13824套，超额完成国家和自治区下达任务，农村危房改造实现竣工7590户。通信基础设施建设取得新成效，行政村宽带覆盖率达100%，城市家庭20兆位/秒及以上宽带接入能力超过98%。物价保持稳定，安全生产形势持续向好，食品药品监管深入开展。民族团结进步创建活动有效实施，圆满完成崇左市庆祝自治区成立60周年活动各项工作，民族团结和宗教界和睦稳定。平安崇左建设不断加强，行政村（社区）实现一村（社区）一法律顾问全覆盖，扫黑除恶专项斗争取得阶段性成绩，禁毒、反恐成效显著，边境立体化防控水平稳步提升，双拥成果进一步巩固发展，全市群众安全感、社会稳定动态八项工作继续排名全区前列。

　　（十）转作风、提效能，政府建设不断加强。坚持以全面从严治党统领政府各项工作。科学拟定改革方案，压茬推进机构改革。深化乡镇机构改革，大力推进“多所合一”。狠抓绩效管理，2017年度我市绩效考评排全区第5名，获得一等等次，取得历史最好成绩。加快社会信用体系建设，公共信用信息平台建成运行。依法接受人大及其常委会监督，自觉接受人民政协民主监督，人大代表建议和政协委员提案办结率100%。全面深化公务用车制度改革，事业单位和国有企业公车制度改革基本完成。改进政府机关作风，广大干部深入一线抓落实、助生产、解难题，政府执行力和公信力不断提升。严格落实中央八项规定精神及实施细则，驰而不息整治“四风”，市本级“三公”经费保持零增长。加强政府系统党风廉政建设，深入推进反腐倡廉，巩固保持了风清气正的良好政治生态。

　　同时，人力资源、侨务、统计调查、审计、法制、计划生育、国有资产监管、海关、海事、新闻出版、广播电视、决策咨询、二轻、供销、涉台事务、机关事务管理、接待、信访、调处、驻邕管理、驻京联络、地震、国家安全、人防、边防、消防、保密、无线电管理、农机、气象、水文、老龄、地方志、档案等工作取得了新进展。银行、保险、邮政、通信、石油、烟草、盐业等行业和工会、共青团、妇联、工商联、科协、文联、侨联、残联、社科联、红十字会、法学会、贸促会等群团组织主动作为、扎实工作、不断进步，为全市经济社会发展作出了积极贡献。

　　各位代表，过去的2018年，我们交出了一张靓丽的成绩单。成绩来之不易，奋斗饱含艰辛。这些成绩的取得，得益于习近平新时代中国特色社会主义思想的科学指引，得益于市委的坚强领导、科学决策，得益于市人大、市政协的有力监督和大力支持，得益于全市人民团结拼搏、共同努力，得益于广大离退休干部亲切关怀和驻崇左人民解放军、武警部队以及社会各界鼎力支持。在此，我代表市人民政府，向全市各族人民，向人大代表、政协委员，向各民主党派、工商联、无党派人士、人民团体，向驻崇左人民解放军、武警部队官兵，向所有关心、支持崇左建设发展的同志们、朋友们，致以崇高的敬意和衷心的感谢！

　　安不忘危、兴不忘忧。在肯定成绩的同时，我们也清醒地看到，我市发展中还存在不少困难和问题。主要是：发展不平衡不充分不协调的问题依然突出，高质量发展的基础还不牢固；传统产业正在深度调整，新动能还接续不上；民生领域还有不少短板，脱贫攻坚依然任务艰巨；政府职能转变还不到位，营商环境需要进一步优化，等等。对此，我们一定高度重视，采取更加有力措施，用新理念、新办法不断解决前进中的各种矛盾和问题，推动经济社会发展行稳致远。

二、 2019年工作安排

　　今年是新中国成立70周年，是全面建成小康社会的关键之年。从外部环境看，改革开放再出发、全面开启新征程，共建“一带一路”正在向落地生根、持久发展的阶段迈进，西部陆海新通道建设进入全新阶段，新经济、新产业、新业态带来创新发展浪潮，为我们全方位扩大开放、促进投资、推动产业转型升级带来了重大机遇、拓展了广阔空间。从发展基础看，经过建市15年的艰苦创业，特别是通过深入实施做好“两篇大文章”、打好“四大攻坚战”三年行动计划，我市经济势能不断释放，开放水平不断提升，产业基础明显夯实，全域旅游成效初显，基础设施不断完善，后发优势日益凸显，为我们在新的起点上推进高质量发展提供了有利条件、增添了更大动力。我们将坚持发展第一要务不动摇，发挥后发优势，抢抓时代机遇，蓄势而为，乘势而上，全面实施做好“两篇大文章”、打好“四大攻坚战”新三年行动计划，推动各项事业进位赶超，实现更高质量、更好效益发展。

　　今年政府工作的总体要求：以习近平新时代中国特色社会主义思想为指导，全面贯彻落实党的十九大和十九届二中、三中全会以及中央、全区经济工作会议、自治区党委十一届五次全会、市委四届五次全会精神，落实“建设壮美广西 共圆复兴梦想”的总目标总要求，持续深入落实“三大定位”新使命和“五个扎实”新要求，坚持稳中求进工作总基调，坚持新发展理念，坚持推动高质量发展，坚持以供给侧结构性改革为主线，坚定不移持续做好“两篇大文章”、打好“四大攻坚战”，统筹推进稳增长、促改革、调结构、惠民生、防风险工作，进一步稳就业、稳金融、稳外贸、稳投资、稳预期，保持经济持续健康发展和社会大局稳定，为与全国全区同步全面建成小康社会打下决定性基础，加快建设祖国南疆繁荣稳定和谐安宁示范区，以优异成绩庆祝中华人民共和国成立70周年。

　　今年经济社会发展主要预期目标：地区生产总值增长8%以上，固定资产投资增长15%，规模以上工业总产值增长17%，规模以上工业增加值增长10%，财政收入增长3%，外贸进出口总额增长4%以上，社会消费品零售总额增长10%，城乡居民人均可支配收入增长8%左右，节能减排控制在自治区下达目标内，城镇登记失业率控制在4.2%以内，居民消费价格指数涨幅3%左右。

　　为实现上述目标，我们将增强崇左自信，保持战略定力，坚持不懈围绕做好“两篇大文章”、打好“四大攻坚战”战略任务，重点抓好以下十个方面工作。

　　（一）大力发展口岸经济，争当沿边开发开放排头兵。主动服务和参与西部陆海新通道建设，深度融入自治区构建“南向、北联、东融、西合”全方位开放新格局，不断拓展开发开放的广度与深度，进一步推动“通道经济”向“口岸经济”转型升级。

　　大力推动西部陆海新通道建设。加强口岸基础设施建设，抓好口岸升格和扩大开放工作，争取凭祥（铁路）口岸获批为进境水果指定口岸、水口口岸扩大开放为国际性口岸通过国家验收、硕龙口岸对外开放通过自治区验收，推动具备条件的边民互市贸易点升级为边民互市贸易区，推动凭祥建设口岸型国家物流枢纽。提升口岸互联互通水平，推进友谊关口岸扩大开放至浦寨通道项目竣工验收，加快建设弄怀—谷楠国际货运专用通道、中越“两国一检”项目；协调建立互市贸易分类通关监管机制，推动具备条件的口岸延长通关时间。

　　更好发挥开放平台作用。用足用活凭祥重点开发开放试验区优惠政策，充分发挥试验区示范引领作用。积极推动跨境劳务合作试点政策落实到位，进一步完善跨境劳务合作管理机制，着力降低企业用工成本。加快建设凭祥综合保税区二期（筹）、凭祥—宁明贸易加工区，大力扶持园区口岸加工、对外贸易壮大发展。

　　推进对外贸易稳量提效。大力发展口岸加工业，推进边民互市贸易转型升级，落实边贸加工扶持政策，促进更多加工企业落地发展。推进中国—东盟（凭祥）进出口水果深加工基地、中泰产业园国际果品加工基地、凭祥东盟特色水果小镇等项目建设，打造坚果食品综合加工和销售基地，促进“边境贸易+落地加工”融合发展。深化跨境经济合作，促进出口加工业大发展。积极争取国家二手车出口试验区落户崇左，打造二手车出口产业链，培育外贸新业态。发挥海峡两岸产业合作区崇左产业园平台作用，用好先行先试政策，引导产业集聚发展。加强对企业规范出口的指导服务，推动外贸自营企业做大做强。

　　（二）加快发展文化旅游，打造国内知名旅游目的地。充分挖掘崇左文化旅游资源，丰富旅游新业态，提升品牌影响力，努力推动“景区旅游”向“全域旅游”“国际旅游”转变，全年接待游客总人数、旅游消费分别突破4000万人次、400亿元。

　　提升文化旅游品牌。加快全域旅游示范区创建工作，推进扶绥县、江州区、天等县创建自治区级全域旅游示范区，力争凭祥市成功创建国家全域旅游示范区。继续推进景区景点建设提升工作，加大景区“创A”力度，建设一批高品质景区，新增5家4A级旅游景区，力争左江花山岩画、友谊关景区创建国家5A级旅游景区景观质量评价报告通过文旅部评审。创新景区运营模式，引进专业团队运营管理景区。实施“旅游饭店建设年”活动，落实扶持政策，引进一批品牌酒店，力争开工建设7—8家四星级以上旅游饭店，推进凯玄国际大酒店、天宝龙大酒店、逸景观岚酒店成功创建四星级旅游饭店。

加快旅游项目建设。加强对明仕田园、人间仙境、都康田园等重点旅游景区景点的规划控制，保护提升田园风光、乡村风貌。加快百里左江生态文化精品旅游带、德天瀑布—花山岩画精品乡村旅游带建设，开发运营好以左江为主线的水上游览项目，推进德天小镇、明仕山庄二期、花山博物馆、上金旅游度假区、龙谷湾文化旅游特色小镇等项目前期工作，开展水口湖国家度假区项目规划编制和招商引资工作。加快民宿项目建设，打造旅游民宿集群。继续深化跨境旅游合作，推动中越德天—板约瀑布跨境旅游合作区实现跨境旅游。

　　丰富文化旅游业态。加快“旅游+”产业融合发展，拓展旅游产品，提升旅游体验。加大花山音画夜游和实景演出营销推广力度，打造文化旅游新爆点。创新发展乡村旅游新产品，创建四星级广西乡村旅游区（农家乐）10家以上。开发多品类系列旅游商品，推广本土特色美食，打造旅游新名片。

　　提高旅游服务品质。加强旅游基础设施建设，新建改建旅游厕所30座，完善全市旅游标识标牌系统，开通吴圩机场、南宁高铁站至崇左的旅游专线车。大力培育和引进旅游专业管理人才，提升旅游服务水平。加强旅游市场监管，维护好旅游市场秩序。

　　拓展文化旅游市场。充分发挥花山岩画世界文化遗产名片、德天跨国瀑布5A级旅游景区和魅力中国城的品牌效应，加强与周边省份及北上广深等目标客源地的宣传推介，深化与东盟国家旅游推广合作，拓宽旅游市场空间。推进崇左智慧旅游建设，开发崇左全域旅游“一张图”“一个APP”，全方位推出崇左旅游资源和旅游攻略，实现“一部手机游崇左”。

　　（三）加快产业转型升级，推进优势产业高质量发展。按照“强龙头、补链条、聚集群”的思路，加快形成传统优势产业改造升级和新兴产业培育壮大齐头并进格局，促进新旧动能转换，提升产业发展质量效益。

　　大力培育龙头企业。推动南国铜业一期年产30万吨电解铜项目全面达产，启动二期项目建设，开工建设川恒集团“铜钛磷”绿色循环产业基地项目，引进一批上规模铜深加工产业项目，培育一批铜业龙头企业，打造千亿元铜业循环经济产业。推进中林集团林产品贸易加工基地、乐林集团高密度纤维板加工、高仁木业板材等项目建设，确保广西德科刨花板等项目竣工投产，加快建设木材交易中心，催生一批林业龙头企业。开工建设华劲纸业竹浆纸一体化等项目，培育新行业龙头企业。

　　着力补短板补链条。推进园区规范化建设，大力实施园区基础设施、配套设施建设攻坚战，加快完善园区水电路、污水处理、工人小区等基础配套设施，新建成标准厂房73万平方米，加快补齐产业支撑平台短板。积极突破企业用电、用工、融资等生产要素瓶颈，加大力度引进一批强优物流企业，提升产业综合配套能力。加快实施糖、锰“二次创业”，拉长产业链条，引进下游新能源专用车等项目，启动湘桂乐斯福活性干酵母二期和酵母抽提物、中信大锰镍钴锰酸锂三元材料项目建设，推动新振锰业锰酸锂等项目建设，确保达利食品、中信大锰锰酸锂技改项目二期、广西中科清能专用电动汽车项目竣工投产。加快发展铝业，争取龙州生态氧化铝项目竣工投产。大力发展稀土产业，推动中铝国盛稀土二期分离、年产5000吨稀土金属项目全面投产，加快建设稀土铝钪合金、稀土氟化物等项目，引进下游稀土永磁电机等项目，促进稀土产业高端化发展。

　　推动形成产业集群。进一步优化产业布局，着力推动木材加工、口岸加工、化学工业等企业数量多、规模相对小的产业协同发展、集聚发展。着力打造龙赞东盟国际林业循环经济产业园、广西山圩产业园、凭祥边境经济合作区林产加工区三个木业加工区。推动广西·中国糖业产业园、中泰产业园金梧桐坚果加工基地、凭祥坚果水果加工基地、龙州水口边贸扶贫产业园做大做强。加快建设中泰产业园绿色表面处理环保科技中心项目，引进一批关联项目，推动海峡两岸产业合作区崇左产业园加快形成产业集聚。推动中小微企业加快成长，培育一批“专精特新”企业。

　　提速发展现代服务业。加快建设华润城南综合体、崇左汽车城、海峡两岸产业合作区崇左产业园无水港作业中心等项目，推进正高国际家居建材市场二期等项目建成运营。推进两岸冷链物流产业合作试点工作，加快建设中国—东盟（凭祥）跨境水产品产业园、中泰产业园国际冷链物流园等项目。大力发展电子商务，推动凭祥综合保税区等开展跨境电商业务，争取创建国家跨境电子商务综合试验区。加快建设凭祥边境贸易服务示范区等服务业集聚区，制定服务业发展优惠政策，培育发展总部经济，打造一批服务业优质企业。

　　创新支撑产业高质量发展。启动数字崇左建设，实施数字政府建设、数字设施升级、数字产业发展、电子商务应用、智慧旅游服务、数字便民平台“六大工程”。落实崇左企业购买科技成果转化补助政策，转化科技成果12项以上。推进政产学研用合作，促进企业加强与中南大学、广西大学等高校及科研院所联合攻关，加速科技成果转化和产业化。加大高新技术企业培育和认定工作力度，高新技术企业保有量30家。加快建设科技企业孵化器、众创空间、工程技术研究中心等科技创新平台，加强关键技术和先进工艺高端化改造。深入实施质量强市战略，积极推动品牌和标准化建设。狠抓商标品牌建设，打造一批具有较强竞争力的崇左品牌。大力实施“人才强市”战略，深入开展“南疆国门聚英才”行动，持续抓好人才政策落实，建强产业人才、乡土人才等人才队伍。研究出台企业高层次人才引进办法。

　　持续强化招商引资。积极扩大与越南、泰国等东盟国家国际产能合作，深化与粤港澳大湾区等区域合作。继续实施“一把手”招商工程，强化产业链招商、以商招商、驻点招商，实现年度区外招商引资项目到位资金363亿元，全口径实际利用外资2.9亿美元。

　　（四）聚焦精准发力，打赢农村全面脱贫攻坚战。脱贫攻坚进入了“啃硬骨头”阶段。按照“核心是精准、关键在落实、确保可持续”要求，深入实施崇左市打赢脱贫攻坚战三年行动计划，抓实乡镇党政正职和专业扶贫工作队两支落实力量，继续打好“五场硬仗”，巩固扶贫成果，不断提升脱贫质量，确保大新县和宁明县顺利脱贫摘帽。计划减贫3.65万人，同时政策兜底1.71万人，71个贫困村出列，天等县完成脱贫摘帽任务。

　　全力攻克脱贫重点难点。继续深化精准帮扶网格化+驻村工作队管理机制，做实做细脱贫攻坚基础工作，强化扶贫信息动态管理，扎实开展集中遍访贫困户活动。继续抓好“种养贸游工”产业扶贫，促进贫困群众稳定增收。发挥贫困村集体经济发展基金引导拉动作用，因地制宜发展乡村旅游、光伏发电等产业，确保已脱贫村和年度预脱贫村集体经济收入达到4万元以上。加快推进易地扶贫搬迁安置点后续管理和旧房拆除等工作，解决好群众搬迁后就业、就学、就医等后续发展问题。完善扶贫项目库建设，加强扶贫项目实施，加快扶贫资金支出。强化扶贫小额信贷贷后跟踪管理，确保贷款“放得出、用得好、收得回”。

　　狠抓深度贫困地区脱贫攻坚。结合1个深度贫困县、7个深度贫困乡镇以及99个深度贫困村的实际问题，加大对深度贫困地区政策倾斜力度，在交通、水利、通信投资项目等方面给予优先安排。引导粤桂扶贫协作资金、项目向深度贫困地区倾斜，不断增强脱贫攻坚合力，坚决打赢深度贫困地区脱贫攻坚战。

加强“扶贫车间”建设。扶持发展种养、加工、旅游、边贸等“扶贫车间”，全力推动“扶贫车间”健康发展。做好“扶贫车间”认证和带头人的信用等级评定工作，规范“扶贫车间”运营管理，切实破解“扶贫车间”融资、用工等难题。

　　全面落实扶贫惠民政策。全面落实教育、卫生、农村危房改造等扶持政策，确保贫困家庭学生“有学上”“上好学”，贫困人口 100%参加城乡居民基本医疗保险、贫困人口住院费用报销比例达90%以上、贫困人口特殊慢性病门诊费用报销比例达 80%以上，加大建档立卡贫困户危房改造、房屋修缮力度，不断提升“三保障”水平。

　　（五）大力实施乡村振兴战略，推进农业农村现代化。树立乡村振兴战略既是民生工程又是经济新增长点的理念，以沿边开放兴边富民工程、驮英水库灌区综合开发工程、乡村生态文化旅游工程、“扶贫车间”脱贫攻坚工程“四大工程”为核心，实施“土地整治+”行动，加快推进农业农村现代化，探索具有崇左特色的乡村振兴之路。

　　提升现代农业发展水平。围绕“稳粮”“强蔗”“兴牧”“优果”思路，加快现代农业发展。落实粮食生产行政首长责任制，年内种植粮食178万亩、产量49万吨以上。稳定甘蔗生产，再建10万亩“双高”基地，全面完成201万亩建设任务。大力发展牛羊和鸽子等特色养殖，新增20个以上牛羊生态养殖小区。加快发展特色水果种植业。推进无公害、绿色、有机农产品生产技术标准种植，新增“三品一标”农产品认证7个以上。加快推进现代特色农业示范区建设，新建成区、县、乡、村四级示范区（园、点）342个。大力培育新型农业经营主体，新增农民专业合作社250家、家庭农场70家、农业企业30家。加快发展农业机械化，推进耕种收综合机械化率达66%，努力提高甘蔗生产机械化水平。加强高标准农田建设，以城乡建设用地增减挂钩为切入点，以扶绥县、江州区为试验区，整县推进“土地整治+”行动，竣工验收“旱改水”项目2万亩，新增水田面积1.4万亩以上。

　　强化乡镇政府能力建设和城乡社区治理。加强乡镇政府公共服务职能建设，扩大乡镇政府服务管理权限，优化乡镇基本公共服务资源配置。推进江州区新和镇经济发达镇行政管理体制改革，充分赋予经济社会管理职能。加强城乡社区基础设施建设，提升城乡社区治理能力。全面开展农村集体产权制度改革。加强农村思想道德建设和公共文化建设，完善村民自治，建立健全村规民约，努力提升乡村社会文明程度。

　　深入推进美丽乡村建设。巩固提升清洁乡村、生态乡村、宜居乡村活动成果，启动实施“幸福乡村”活动，打造“美丽崇左”乡村建设升级版。开展农村人居环境整治三年行动和乡村风貌提升三年行动，启动扶绥县、江州区全域实施乡村风貌提升示范县（区）工程，完成100个“四个好”幸福乡村建设和10个星级精品村示范创建工作。

　　（六）加快新型城镇化建设，推动城乡区域协调发展。围绕建设山水园林城市目标，加快新型城镇化建设步伐，促进城乡一体化发展，全市城镇化率提升到42.5%。

　　全面提升中心城区品位。坚持规划引领作用，高水平编制各片区控制性详细规划，力争实现控规100%全覆盖。推进中心城区大投入大建设，继续加快七大系列项目建设，建成区面积增加4平方公里。实施中心城区提档升级工程，开业运营爱琴海购物公园、太平古城一期，开工建设崇左高铁站片区基础设施和配套设施项目。加强城区路网建设，建成使用明仕大道、崇善大道、江南路西段改造等项目，加快建设环城北路等项目，开工建设友谊大桥、丽江大桥。进一步完善城区配套设施，开工建设城区生态水系修复工程、崇左市生活垃圾焚烧发电项目，加快建设江北污水处理厂、中心城区地下缆线管廊等项目。继续推进国家园林城市建设，市区新增绿地面积80公顷以上。

　　加强县城和特色城镇建设。加快发展县域经济，努力打造扶绥县南宁卫星城、大新县全国土司文化之城、天等县农民工创业之城、宁明县花山之城、龙州县天琴之城、凭祥市东盟之城。加快推进凭祥市中小城市综合改革先行先试，巩固提升大新县新型城镇化示范建设成果。狠抓特色城镇建设，力争山圩木艺小镇、水口军民融合边贸特色小镇纳入全国特色小镇建设范围，新和甜蜜小镇通过全国特色小镇验收命名。

　　提升城市管理水平。深入开展自治区文明城市创建工作。落实城市管理执法体制改革，加快建立完善长效管理机制。巩固和加强市容环境综合整治。运用信息化手段、实施网格化管理，不断提升城市管理水平，塑造南疆国门城市新形象。

　　（七）强化基础设施建设，增强发展保障能力。坚持基础设施先行，加快左右江革命老区振兴规划建设，下更大的力气补齐基础设施短板，为经济社会发展提供有力支撑。

　　以补短板为重点全力扩大投资。坚持以“双10”项目建设为抓手，全力推进市级层面以上统筹推进的280项重大项目建设，集中力量狠抓项目储备、落地开工、竣工投产等关键环节，确保完成投资300亿元以上。高质量做好项目前期工作，继续开展项目并联审批办法，推行项目审批容缺受理机制，完善项目推进高效机制。规范实施PPP项目，重点实施龙赞东盟国际林业循环经济产业园（一期）、中泰产业园国际果品加工基地、凭祥—宁明贸易加工区产业大道项目。加强项目融资工作，深化政银企对接，多渠道解决融资难题。

　　加快建设现代交通体系。加快建设南宁至崇左城际铁路，争取启动项目延伸至凭祥前期工作，推进湘桂铁路南宁至凭祥段货运通道规划与建设。竣工通车崇左至水口高速公路、大新桃城至龙州金龙二级公路大新段等项目，加快建设隆安至硕龙高速公路、大新德天至宁明花山公路、山圩至中泰产业园一级路等项目，开工建设崇左至爱店、吴圩机场经扶绥至隆安高速公路，推进崇左至靖西延伸至硕龙高速公路、国道219沿边公路（崇左段）前期工作，启动田东经天等至大新高速公路、大新经龙州至凭祥高速公路前期工作。加强水运基础设施建设，开工建设山秀船闸扩能工程。加快建设工业园区、边贸口岸基础配套路网工程，促进园区、口岸与地方经济融合发展。

　　加强水利基础设施建设。扎实做好左江治旱黑水河现代化灌区工程等项目前期工作，加快建设驮英水库及灌区工程、左江崇左城区河段防洪治理工程等项目。继续做好冬修水利、中央财政小农水项目、病险水库除险加固、中小河流治理和水土保持等工作。

　　抓好民生基础设施建设。加快“四好农村路”建设，力争创建1个以上自治区级示范县。推动大新县、宁明县通天然气，实现“县县通天然气”。加强电网建设，新建220千伏宁明送变电工程，建成投产110千伏龙源（山圩）送变电工程等一批主电网和农配网项目。新建340个4G基站，解决边境地区4G信号弱问题。

　　（八）坚持绿色发展，加快建设美丽崇左。践行绿水青山就是金山银山的理念，进一步厚植生态优势，坚持绿色发展，加快创建山清水秀生态文明示范市。

　　打好污染防治攻坚战。深入实施大气、水、土壤、生态环境保护基础设施建设等4个三年攻坚行动，认真抓好中央环境保护督查“回头看”整改工作，坚决守住生态红线和环保底线。加强道路工地扬尘、餐饮油烟污染、露天焚烧垃圾、秸秆焚烧等专项治理，逐步推行烟花爆竹禁燃限放，确保空气质量保持在全区前列。加强源头监管，治理土壤和固体废弃物污染。严格落实“河长制”，完成县级集中式饮用水水源地环境问题治理工作，确保地表水和市县集中式饮用水水质100%达标。

　　加强生态保护与建设。全面实施国土绿化扩面提质行动，深入推进石漠化综合治理，巩固退耕还林成果，加强自然保护区建设管理，完成植树造林13万亩、中幼林抚育21.5万亩。深入实施山水林田湖草生态保护修复工程试点工作，建成一批国家试点工程示范项目，确保通过2019年国家中期考核评估。深入实施“林长制”，全面推进集体林地林权证发放和不动产登记，开展国有林场林地被侵占综合整治工作，积极兑现森林生态效益补偿基金，开展森林资源规划设计调查。健全生态环境保护责任体系，继续推进领导干部自然资源资产管理责任审计工作。

　　推进资源节约和循环利用。建立健全资源总量和全面节约制度。加强绿色生产政策引导，推进农药、化肥零增长行动和生物防治病虫害，培育节能环保、清洁能源产业。

　　（九）持续优化营商环境，打造发展新优势。以解放生产力、提高竞争力为目标，一切以群众、企业、客商、项目为中心，启动“流程再造、服务重塑”工程，深入推进体制机制改革，全力打造效率更高、服务更优的一流营商环境。

　　深化“放管服”改革。深入推进简政放权，深化“一事通办”改革，年内实现90%以上的依申请行政权力事项和公共服务事项办理“最多跑一次”。全面推进“互联网+政务服务”，实现政务服务事项“应上尽上、全程在线”。实施财税优惠政策兑现“快车道”审批办法，设立专门窗口受理企业奖补申请。深化商事制度改革，全面推进“证照分离”改革，持续推进“多证合一”动态调整工作。

　　提高服务企业成效。完善服务企业工作机制，持续开展“市长服务企业日”活动，实施规模以上企业服务专员制度，推进市县两级服务企业机构建设，引入第三方机构开展服务企业评价考核。完善市服务企业综合信息平台功能，扩大市场主体覆盖范围。加快推进企业上限上规入统工作，新增工业企业入统40家、服务业企业入统50家。毫不动摇支持民营企业发展，全面兑现优惠政策承诺，集中梳理、解决“新官不理旧账”等失信行为；着力治理公路乱收费、乱罚款等问题，降低企业生产经营成本；加快健全政府性融资担保体系，鼓励和支持金融机构加大信贷投放，缓解民营企业融资难融资贵问题；积极主动服务好民营企业发展，保护好民营企业家人身安全和财产安全，构建“亲”“清”新型政商关系。

　　深化重点领域改革。稳妥有序推进机构改革。按照“巩固、增强、提升、畅通”八字方针，持续深化供给侧结构性改革。积极参与面向东盟的金融开放门户建设，推动创建保险创新综合试验区，持续提升跨境人民币结算业务水平；加强金融基础建设，丰富金融体系，大力支持企业上市，设立政府引导基金和产业投资基金，提升金融服务实体经济能力；加大“引金入崇”力度，年内引进桂林银行设立崇左分行。深入开展互联网金融风险专项整治，妥善处置不良贷款，有效防控金融风险。依法依规加快推进制糖企业战略重组。深化电力体制改革，推动更多企业纳入全区电力直接交易范围，推进跨合能源公司实现开展配电增值业务。加快园区体制改革，全面实施中泰产业园代管渠旧镇、濑湍镇。深化国企国资改革，推动市城建集团实现AAA信用等级、市工投集团实现AA信用等级、左江金土公司实现A信用等级。统筹推进财税、文化、供销等各领域改革，推动各项改革向纵深发展。

　　（十）坚持以人民为中心，提高保障和改善民生水平。紧盯老百姓最关心最直接最现实的利益问题，一件接着一件办，一年接着一年干，踏踏实实解民忧、惠民生、增福祉，不断满足人民群众对美好生活的需求。

　　优先发展教育事业。多渠道筹措资金，新建、迁建和改扩建一批学校。加强中心城区学校建设，开工建设城南实验学校，推进广西理工职业技术学院城北校区、广西外国语学院和江州高中等项目建设，完成崇左高中、城南小学改扩建工作。大力发展学前教育，加快公办幼儿园建设，扶持多元普惠幼儿园发展。实施新一轮“基础教育质量提升工程三年行动计划”，深入实施名校、名师和名校长“三名”工程，推动义务教育从基本均衡向优质均衡升级。支持发展高等教育，推进广西城市职业学院职业教育本科试点工作，完成南宁地区教育学院转制工作，支持广西民族师范学院内涵建设发展。加强教师队伍建设，实施教师交流轮岗、支教走教计划，着力缓解乡村教师短缺问题。加强校园信息化建设。抓好民办学校管理。

　　加快健康崇左建设。深入推进国家卫生城市创建工作。深化医药卫生体制改革，推进公立医院综合改革，加快建立现代医院管理制度。规范医联体建设，推进分级诊疗。加强医疗卫生服务体系建设，推进市人民医院成功创建三级甲等医院，开工建设一批基层医疗卫生机构能力建设项目。大力发展康养产业，推进康养小镇建设。实施卫生人才强基工程，落实全科医生转岗培训计划和乡村医生培训计划，着力缓解基层卫生专业技术人员短缺问题。加强疾病预防控制，加快自治区健康促进县及自治区级慢性病防控综合示范区建设。全面优化计划生育服务管理。实施食品安全战略，推进广西食品安全示范城市创建工作。

　　加强就业和社会保障。落实就业优先政策，新增就业1.4万人以上，新增农村劳动力转移就业3.2万人以上。继续推进“全民参保登记计划”，加强社会保险扩面征缴，完善社会救助体系。继续加强物价调控，做好保供稳价工作。稳步推进保障性住房建设，棚户区改造开工15258套。

　　大力发展文化体育事业。继续开展“三月三”系列活动。加强花山文化精品创作，推动“天琴艺术”“高腔诗雷”申报国家级非物质文化遗产。抓好一批村级公共服务中心建设。深入开展文化体育活动，举办全市群众文艺汇演和崇左市第一届农民篮球赛，开展一批具有边关特色的体育活动。办好龙州起义90周年纪念活动。

　　全力维护边疆安全稳定。全面开展建设祖国南疆繁荣稳定和谐安宁示范区活动，深入推进扫黑除恶专项斗争，加快智慧政法建设步伐，发挥“三个一”工作机制作用，深入排查化解社会矛盾纠纷，进一步提升全市公众安全感和满意度。强化边境管控，加快建设边境综合拦阻监控设施，维护好边境安全稳定。加强安全生产监管，铁腕整治各类风险隐患，坚决遏制安全事故发生。

　　实施为民办实事工程。筹措资金45亿元以上，实施社保、健康、教育、水利、安居、农补、生态、文化、扶贫、科技等十大类惠民项目。

　　加强民族宗教工作。认真贯彻落实民族宗教政策，深入开展民族团结进步创建活动，大力实施兴边富民行动，筑牢民族团结生命线。

　　支持国防和军队建设。加强全民国防教育，深化国防动员、国防后备力量和人民防空建设。推进军民融合深度发展，积极创建自治区和全国双拥模范城。

三、 全面加强政府自身建设

　　跨入新起点，形势逼人；接力新征程，重任在肩。我们将在市委的正确领导下，进一步加强法治政府、诚信政府、高效政府、廉洁政府、服务型政府、创新型政府建设，不断提高政府治理能力和服务水平，努力建设人民满意的政府。

　　（一）切实提高政治站位。坚持以党的政治建设为统领，牢固树立“四个意识”，增强“四个自信”，坚决维护习近平总书记核心地位，坚决维护党中央权威和集中统一领导，在政治立场、政治方向、政治原则、政治道路上同以习近平同志为核心的党中央保持高度一致。不折不扣贯彻落实中央、自治区的决策部署，不折不扣坚决执行市委的决议决定，自觉做到政令畅通。

　　（二）勤于学习勇于创新。扎实推进“两学一做”学习教育常态化制度化，开展“不忘初心、牢记使命”主题教育，深化政府系统意识形态建设，坚持用习近平新时代中国特色社会主义思想武装头脑、指导实践、推动工作。主动适应高质量发展新要求，持续深化专业知识学习，切实增强“八个本领”，努力提升政府工作水平。坚持解放思想，努力破除一切影响制约发展的旧思想、旧观念，形成鼓励大胆探索、勇于创新的良好氛围。

　　（三）坚持推进依法行政。深入落实法治政府建设实施纲要，加快构建系统完备、科学规范、运行有效的依法行政制度体系，确保政府工作始终在法治轨道上运行。自觉接受市人大法律监督、工作监督和市政协民主监督，主动接受市监察委依法监察，高质量做好人大代表议案、建议和政协委员提案办理工作。加强招商引资、招投标、政府债务等重点领域政务诚信建设，坚决履行依法作出的各项政策承诺。

　　（四）始终坚持实干担当。加强政府执行能力建设，强化担当意识，带头把责任扛在肩上，用90%以上的时间和精力抓落实，确保各项部署落地生根。加强专业能力建设，用专业思维、专业方法将工作落到实处。加强政务督查并强化结果运用，优化完善奖惩机制，提高绩效考评指标体系的针对性和准确性。健全正向激励和容错纠错机制，旗帜鲜明地为担当者担当、对负责者负责，推动形成想作为、敢作为、善作为的良好风尚。

　　（五）转变作风提升效能。着力推进作风建设常态化、制度化、长效化，整治消极懈怠不担当等不良作风，特别在机构改革期间，切实履好职、尽好责，确保做到思想不乱、队伍不散、工作不断、劲头不减，各项工作平稳有序过渡。牢固树立“服务就是义务、服务就是环境、服务就是效益”理念，积极践行“一线工作法”，把握企业需求、基层呼声和群众期盼，用心当好服务企业、服务项目、服务群众的“店小二”，以优质的服务赢得群众的口碑。实施群众和全媒体问政工作，自觉接受群众监督，积极回应群众关切。全面落实政务和信息公开制度，进一步加强公众参与平台建设，畅通民意反映渠道，让人民监督权力，让权力在阳光下运行。

　　（六）加强勤政廉政建设。认真落实政府系统全面从严治党主体责任，严格执行《中国共产党廉洁自律准则》《中国共产党纪律处分条例》，严守党纪国法红线、廉洁从政防线、道德操守底线，把权力关进制度的笼子。认真落实中央八项规定精神及实施细则，严格执行自治区党委、政府和市委各项规定，坚持不懈纠正“四风”。加强工程建设、土地出让、政府采购、公共资源交易等重点领域监管，强化财政检查和审计监督，认真抓好巡视巡察反馈问题整改，狠抓扶贫领域腐败和作风问题专项治理，坚定不移推进政府系统廉政建设和反腐败工作，持之以恒正风肃纪，以干部清正、政府清廉、政治清明取信于民。

　　各位代表，使命呼唤担当，奋斗铸就辉煌！让我们紧密团结在以习近平同志为核心的党中央周围，以习近平新时代中国特色社会主义思想为指导，在市委的坚强领导下，不忘初心、牢记使命，开拓进取、奋勇争先，坚定不移做好“两篇大文章”、打好“四大攻坚战”，加快建设祖国南疆繁荣稳定和谐安宁示范区，以优异成绩庆祝中华人民共和国成立70周年，为“建设壮美广西、共圆复兴梦想”作出更大贡献！
